# Association between non-alcoholic fatty liver disease and ischemic stroke

**Published:** 2014-07-04

**Authors:** Hanieh Moshayedi, Reza Ahrabi, Afshin Mardani, Saieed Sadigetegad, Mehdi Farhudi

**Affiliations:** 1 Department of Neurology, School of Medicine, Tabriz University of Medical Science, Tabriz, Iran; 2 Department of Radiology, School of Medicine, Tabriz University of Medical Science, Tabriz, Iran; 3 Department of Neurology, School of Medicine, Neurosciences Research Center, Student Research Committee, Imam Reza Hospital, Tabriz University of Medical Sciences, Tabriz, Iran

**Keywords:** Non-alcoholic Fatty Liver Disease, Carotid Intima-media Thickness, Ischemic Stroke

## Abstract

**Background: **Some studies in recent years showed that carotid intima-media thickness (IMT), indicator of the presence of atherosclerosis, was higher in non-alcoholic fatty liver disease (NAFLD) in comparison with normal subjects. They concluded that NAFLD patients may be resulted in more cardiovascular events. Hence, we aimed to study the association of NAFLD and ischemic stroke.

**Methods:** For this reason, 110 brain magnetic resonance imaging confirmed ischemic stroke patients and 110 patients age and sex matched controls went through liver ultrasound to detect NAFLD and common carotid ultrasound to measure IMT. Demographic and vascular risk factors were detailed for all subjects.

**Results: **NAFLD was found in 47 (42.7%) of ischemic stroke patients and 25 (22.7%) of controls. By adjusting sex and age in table 2, odds ratio (OR) for NAFLD was 2.15 (95% confidence interval [CI]: 1.25-3.71) that was statistically significant (P = 0.006). However, after adjusting for other confounding risk factors (waist circumference, hypertension, diabetes mellitus, low-density lipoprotein, triglyceride, alanine aminotransferase, aspartate aminotransferase, creatine, body mass index, cigarette smoking, and ischemic heart disease), the OR decrease to 1.68 (95% CI: 0.42-6.76) that was not statistically significant (P = 0.460). The OR for IMT of right and left common carotid was 1.23 (95% CI: 0.48-3.15) and 1.24 (95% CI: 0.57-2.69), respectively that none of them were statistically significant.

**Conclusion: **Although the risk of occurrence of ischemic stroke is higher in NAFLD patients, but NAFLD is not associated independently with ischemic stroke.

## Introduction

Incidence of stroke in Iran estimated as 72,100-100,000 case/year. In comparing with developed countries not only, the incidence rate is higher, but also the onset age of stroke is a decade earlier in our country.^1^^,^^2^ As ischemic stroke is an incurable disease in most cases, the prevention strategies play a vital role in managing of patients at risk.

Non-alcoholic fatty liver disease (NAFLD) frequency ranges from 9% to 36.9% of the population in different parts of world^3^^-^^5^ and in a recent study it is estimated as 21.5% in Iranian adult general population.^6^ NAFLD is a cause of fatty liver, occurring when fat is deposited (steatosis) within the hepatocytes in people with no history of excessive alcohol consumption.^7^ The best diagnostic test for confirming NAFLD is liver biopsy,^8^ but except in patients with progressive fatty liver diseases, its use has been limited because of medical and ethical considerations.^9^^,^^10^ Elevated levels of liver enzymes, such as aspartate aminotransferase (AST) and alanine aminotransferase (ALT) are common laboratory abnormalities found in patients with NAFLD, but the specificity of these tests is low.^9^ Consequently, the clinical evaluation of NAFLD is commonly based on a combination of ultrasonographic findings and laboratory tests.^11^

The intima-media thickness (IMT) of the carotid artery is a reliable indicator of subclinical atherosclerosis and studies have showed that the increased IMT associated by increased prevalence and incidence of myocardial infarction and stroke.^12^ B-mode carotid duplex scanner allows for direct visualization of the arterial walls. In recent years, carotid artery IMT measurements have been widely used to study the severity and progression of atherosclerosis.^13^

In recent years, some studies^14^^-^^18^ investigated the relationship between NAFLD and cardiovascular events and showed that carotid IMT was higher in NAFLD than normal subjects. Hence, they concluded that NAFLD may be a leading factor in atherosclerosis formation and may result in more cardiovascular events.

As the frequency of NAFLD is considerable in all the world^3^^-^^6^ and predicted to increase in the future because of the growing number of obese individuals, so detecting and treatment of NAFLD in early stages by weight loss and exercise^19^ would be beneficial in prevention of ischemic stroke.

The main aim of the present study was to evaluate the relationship between NAFLD and ischemic stroke, to realize whether NAFLD is a direct leading factor in occurrence of ischemic stroke or not. In addition, we aimed to investigate the association between common carotid IMT and ischemic stroke.

## Materials and Methods

This cross-sectional study was performed at Razi and Imam Reza University Hospitals of Tabriz University of Medical Science, Tabriz, Iran between May 2012 and November 2013. This study was approved by Tabriz University of Medical Science Local Ethics Committee (Approval Number: 5/4/1681) and informed written consents were provided for all participants. 

Sample size, because of lack of a similar study as a model, estimated based on the number of parameters of the regression model as 220 subjects. Case group consisted of 110 ischemic stroke patients that all were evaluated by computed tomography scan and magnetic resonance imaging (diffusion weighted) to verify ischemic stroke as a sole pathological cause of patients’ clinical presentations and to exclude other possible pathologies such as tumor, hemorrhage or previous ischemic stroke. Controls include 110 age and sex matched individuals without history of stroke.

All participants interviewed, and medical history of alcoholic consumption, smoking, viral or autoimmune hepatitis, using any hepatotoxic drugs, diabetes mellitus (DM), hypertension (HTN), and ischemic heart disease (IHD) were obtained. Chronic hepatitis B and C serologically excluded. Persons with history of alcohol consumption more than 20 g/day and chronic hepatotoxic drug users were also excluded.

Weight and height were measured and body mass index (BMI) was calculated as weight divided by height squared (kg/m^2^). Waist circumference (WC) was measured in a standing position at the midway between the lowest rib and the iliac crest.

Resting blood pressure was measured 3 times and the mean of second and third ones was considered. After 8 h fasting blood samples were analyzed for low-density lipoprotein (LDL), triglyceride (TG), ALT, AST, creatine (Cr), and fasting blood sugar.

Liver ultrasound scans were performed for all subjects to evaluate the presence and severity of NAFLD. All liver ultrasound scans were done by a single operator with B mode scanner (SSD-5500, Aloka, Tokyo, Japan). The grading of NAFLD assessed on the basis of increased echogenicity and possibility of visualization of the diaphragm and intrahepatic vessel border. NAFLD grading described as G0 (absent), G1 (Slightly increased echogenicity, with normal visualization of the diaphragm and the intrahepatic vessel borders), G2 (Moderately increased echogenicity, with slightly impaired visualization of the diaphragm and the intrahepatic vessel borders) and G3 (Severely increased echogenicity, with poor or no visualization of the diaphragm and the intrahepatic vessel borders, and posterior portion of right lob).^20^

The IMT (The distance between the leading edge of the first and second echogenic lines) was measured at the far wall of both common carotid arteries approximately 2, 1, and 0.5 cm proximal to the carotid bulb at a site free from any discrete plaque. The carotid IMT was defined as the mean of the maximal IMT of three measurements of each common carotid artery. A single examiner took all measurements.

All data put in (SPSS) for Windows 19.0 (SPSS Inc., Chicago, IL, USA) and analyzed through descriptive statistics and Student’s t-test. Furthermore, data analyzed by Stata statistical software through conditional logistic regression models to estimate odds ratios (ORs) and 95% confidence intervals (CIs). To evaluate the independent association of ischemic stroke and NAFLD, the first model adjusted for age and sex and the second model additionally adjusted for DM, HTN, IHD, AST, ALT, LDL, TG, BMI, Cr, WC, and smoking. To determine the association between NAFLD and carotid IMT in ischemic stroke patients the multiple linear regression analyses were performed separately in subjects with and without NAFLD in the case and control groups. P <0.05 considered to be significant.

## Results

The frequencies of age, sex, BMI, NAFLD, IMT, AST, ALT, WC, LDL, high-density lipoprotein, TG, DM, IHD, HTN, Cr, and smoking at both groups (cases and controls) were summarized in table 1. 

NAFLD was found in 47 (42.7%) of ischemic stroke patients and in 25 (22.7%) of controls. It showed that the frequency of NAFLD was significantly higher in ischemic stroke patients (P = 0.001).

The mean of ALT and AST were significantly higher in cases in comparison with controls (P < 0.001). Even though, the mean of carotid IMT were slightly higher in ischemic stroke patients in comparison with controls, but the association was not statistically significant (P = 0.09).

By adjusting sex and age, OR for NAFLD was 2.15 (95% CI: 1.25-3.71) that was statistically significant (P= 0.006). It demonstrated NAFLD increase the risk of ischemic stroke 2.15 times in comparison with sex- and age-matched population.

However, when we matched other confounding factors (WC, HTN, DM, LDL, TG, AST, ALT, Cr, BMI, cigarette smoking, and IHD) the OR reduced to 1.68 (95% CI: 0.42-6.76). Although, it showed that NAFLD increased risk of ischemic stroke 1.68 times in comparison of persons with the same risk factors, it was not statistically meaningful (P = 0.460).

All NAFLD were estimated in grade 1, so the evaluation of ultrasound grading of NAFLD in ischemic stroke patients could not be possible in this study.

OR for ALT was 1.04 (95% CI: 1.01-1.06) that was statistically significant risk factor at age and sex matched model (P = 0.001), but not meaningful at multivariate matched model (P = 0.352). No significant differences were seen for AST at both models.

The OR for IMT of right and left common carotid were 1.23 (95% CI: 0.48-3.15) and 1.24 (95% CI: 0.57-2.69), respectively that none of them were statistically significant. Regarding these results increased IMT might not associated by higher risk of ischemic stroke.

**Table 1:** The clinical and biological characteristics of the participants

**Characteristics**	**Cases (n = 110)**	**Controls (n = 110)**	**P**
Age	66.42 ± 11.31	66.51 ± 11.27	NS
Sex (F/M)	41/69	41/69	NS
NAFLD (%)	42.7	22.7	0.001
BMI (kg/m^2^)	26.71 ± 3.02	25.34 ± 2.65	NS
Waist circumference (cm)	106.79 ± 97.37	91.67 ± 9.24	0.020
Smoking (%)	50	49	NS
DM (%)	50.90	11.82	0.000
HTN (%)	66.36	39.09	0.000
TG (mg/dl)	217.00 ± 90.23	174.00 ± 65.31	0.001
LDL (mg/dl)	221.28 ± 70.09	162.72 ± 46.90	0.010
HDL (mg/dl)	48.83 ± 12.01	52.63 ± 12.81	NS
Cr (mg/dl)	1.14 ± 0.36	1.03 ± 0.18	0.000
ALT (IU/L)	30.52 ± 13.88	24.20 ± 10.85	0.000
AST (IU/L)	26.94 ± 14.25	23.68 ± 9.18	0.000
RCC-IMT (cm)	1.05 ± 0.29	1.03 ± 0.33	NS
LCC-IMT (cm)	1.08 ± 0.36	1.06 ± 0.37	NS

NS: Non-significant; NAFLD: Non-alcoholic fatty liver disease; BMI: Body mass index; DM: Diabetes mellitus; HTN: Hypertension

TG: Triglyceride; LDL: Low-density lipoprotein; HDL: High-density lipoprotein; Cr: Creatine; ALT: Alanine aminotransferase

AST: Aspartate aminotransferase; IMT: Intima-media thickness; RCC: Right common carotid; LCC: Left common carotid

**Table 2:** Association between non-alcoholic fatty liver disease and ischemic stroke

**Characteristics**	**Age- and sex-matched**		**Multivariate matched**	
	**OR (95% CI)**	**P**	**OR (95% CI)**	**P**
NAFLD	2.15 (1.25-3.71)	0.006	1.68 (0.42-6.76)	0.460
ALT	1.04 (1.01-1.06)	0.001	1.02 (0.96-1.09)	0.352
AST	1.01 (0.99-1.04)	0.069	0.93 (0.87-1.00)	0.089

OR: Odds ratio; CI: Confidence interval; NAFLD: Non-alcoholic fatty liver disease; ALT: Alanine aminotransferase;

AST: Aspartate aminotransferase

## Discussion

Our study showed that the frequency of NAFLD was higher in ischemic stroke patients in comparison of controls. The OR of 2.15 demonstrated that NAFLD considerably increased risk of ischemic stroke as compared with normal age and sex matched individuals. The means of liver function tests in our cases were higher than controls and OR of 1.04 (95% CI: 1.01-1.06) for ALT depicted that it was statistically correlated with greater risk of ischemic stroke (P = 0.001). However, similar results were not found for AST.

Our findings were roughly consisted with the results of cross-sectional records-based study that Ivan Ying et al.^21^ conducted. They included 103 ischemic stroke and 200 controls. The adjusted OR for acute ischemic stroke in the presence of an elevated ALT was 3.3 (95% CI: 1.3–8.4). Similar elevations were obtained for AST concentration. They concluded that NAFLD increases ischemic stroke risk 3.3 times.

Ying et al.^21^ just relied on liver function tests as a diagnostic tool of NAFLD, however in some NAFLD patients the level of AST and ALT are in normal range, and liver function tests may be neither sensitive nor specific in diagnosis of NAFLD,^14^ so the accuracy of their results might be doubted. The diagnosis of NAFLD in our study was based on ultrasound findings and the exclusion of other common causes of fatty liver in addition of liver function tests. The gold standard of diagnosis of NAFLD is liver biopsy, but its use limited to progressive fatty liver diseases because of medical and ethical considerations.^9^^,^^10^ Consequently, the clinical evaluation of NAFLD is commonly based on a combination of ultrasonographic findings and laboratory tests.^11^

After adjusting for major confounding factors (DM, HTN, IHD, AST, ALT, LDL, TG, BMI, Cr, WC, and smoking) the OR of NAFLD reduced to 1.68 (95% CI: 0.47-06.76). These parameters (DM, HTN, IHD, …) are the known main leading factors of ischemic stroke.^22^ The OR of 1.68 depicted greater risk of ischemic stroke in individuals with NAFLD, but statistically there was no independent relationship between NAFLD and ischemic stroke (P = 0.460). So, our study showed the confounding effects of known cardiovascular risk factors would not be negligible in relation of NAFLD and ischemic stroke.

Our findings were consistent with the results of Kim et al.^18^ study. They evaluated the association of NAFLD and carotid IMT by ultrasound among 556 men and 465 women. Their study showed that persons with NAFLD had greater carotid IMT than subjects without NAFLD. However, the difference in IMT was statistically meaningful only in subjects with risk factors of DM, HTN, hyperlipidemia, and obesity.

The mean of carotid IMT in our study were slightly higher in ischemic stroke patients than controls; however, statistically meaningful differences were not found. These results were consistent with findings of Petit et al.^23^ study that showed steatosis (liver fat) did not correlate with IMT. Although, some studies^14^^-^^18^ showed that NAFLD is strongly associated with carotid IMT, which is in contrast of our study. This inconsistency may be due to small sample size of our study and also the study design differences that we did not evaluate IMT in NAFLD patients as previous studies on the contrary we evaluated IMT in ischemic stroke patients.

To confirm the association of NAFLD and ischemic stroke, we suggest conducting a cohort study that NAFLD diagnosed patients to be followed-up regarding developing ischemic stroke.

However, by having present time’s evidences and considering great prevalence of NAFLD in the normal population,^3^^-^^6^ it seems to be beneficial to diagnose NAFLD by liver ultrasound and liver function tests in patients with ischemic stroke risk factors.

## Conclusion

This survey showed that NAFLD is much more prevalent in ischemic stroke patients than the healthy population. In addition, the risk of ischemic stroke is higher in patients with risk factors of NAFLD.

## Notes

**How to cite this article: **Moshayedi H, Ahrabi R, Khoshbaten M, Tarzemani MK, Hashemilar M, Masudian N, et al. Association between non-alcoholic fatty liver disease and ischemic stroke. Iran J Neurol 2014; 13(3): 144-8.
